# Comparison of GlideScope Videolaryngoscopy to Direct Laryngoscopy for Intubation of a Pediatric Simulator by Novice Physicians

**DOI:** 10.1155/2013/407547

**Published:** 2013-10-31

**Authors:** Joni E. Rabiner, Marc Auerbach, Jeffrey R. Avner, Dina Daswani, Hnin Khine

**Affiliations:** ^1^Department of Pediatrics, Division of Pediatric Emergency Medicine, Children's Hospital at Montefiore, Albert Einstein College of Medicine, 3315 Rochambeau Avenue, Bronx, NY 10467, USA; ^2^Department of Pediatrics, Division of Pediatric Emergency Medicine, Yale School of Medicine, 100 York Street, Suite 1F, New Haven, CT 06511, USA

## Abstract

*Objective*. To compare novice clinicians' performance using GlideScope videolaryngoscopy (GVL) to direct laryngoscopy (DL). *Methods*. This was a prospective, randomized crossover study. Incoming pediatric interns intubated pediatric simulators in four normal and difficult airway scenarios with GVL and DL. Primary outcomes included time to intubation and rate of successful intubation. Interns rated their satisfaction of the devices and chose the preferred device. *Results*. Twenty-five interns were included. In the normal airway scenario, there were no differences in mean time for intubation with GVL or DL (61.4 versus 67.4 seconds, *P* = NS) or number of successful intubations (19 versus 18, *P* = NS). In the difficult airway scenario, interns took longer to intubate with GVL than DL (87.7 versus 61.3 seconds, *P* = 0.018), but there were no differences in successful intubations (14 versus 15, *P* = NS). There was a trend towards higher satisfaction for GVL than DL (7.3 versus 6.4, *P* = NS), and GVL was chosen as the preferred device by a majority of interns (17/25, 68%). *Conclusions*. For novice clinicians, GVL does not improve time to intubation or intubation success rates in a pediatric simulator model of normal and difficult airway scenarios. Still, these novice clinicians overall preferred GVL.

## Introduction

Successful laryngoscopy and tracheal intubation are crucial skills necessary for management of the airway in critically ill infants and children. Proficiency in these skills requires training, practice, and experience. The GlideScope videolaryngoscope (Verathon Medical, Bothell, WA, USA) has been developed to facilitate tracheal intubation, especially in difficult airway scenarios, by providing a wider angle, unobstructed view of the glottis [1–5]. The GlideScope has been shown to provide a view of the larynx that is as good as, or better than, standard direct laryngoscopy [4, 6–9] and does not require oral, pharyngeal, and tracheal axis alignment for intubation. Additionally, the GlideScope allows the trainee and the supervisor to view the same image concurrently on the video screen. 

Previous studies comparing GlideScope videolaryngoscopy (GVL) to direct laryngoscopy (DL) in terms of ease of use and time to intubation among experienced clinicians have reported conflicting results in both adult and pediatric populations. In pediatric studies comparing time to intubation between these devices in the operating room when used by experienced anesthesiologists, some have found that GVL required a longer time [10, 11] while others have found no difference in time to intubation between GVL and DL [12, 13]. Another pediatric study found no difference in time to intubation between the two devices when used by experienced anesthesiologists or intensivists to intubate pediatric manikins [14]. 

Among the studies utilizing novice clinicians, the findings were similarly varied. A study using paramedics, medical students, respiratory therapists, and nurses in the real live situation of the operating room showed that GVL has higher success rates and shorter times to intubation for adult patients [15]. Using a neonatal normal airway manikin model, another study found prolonged times to intubation with GVL when performed by medical and nursing students [16]. However, there have been no studies to date examining the ease of operation of the GVL by untrained, novice medical providers on a pediatric model with both normal and difficult airway scenarios.

The objective of this study was to compare the performance of novice clinicians in intubating a pediatric model using GVL to the standard DL, using a Miller laryngoscope blade. We hypothesized that the GlideScope intubation technique would be superior to DL in terms of intubation success rates and time to intubation for novice physicians in both normal and difficult airway scenarios and that the novice physicians would prefer GVL over standard DL.

## 2. Materials and Methods

### 2.1. Study Design

This was a prospective, randomized crossover study conducted at an urban, quaternary care children's hospital. Postgraduate year one pediatric residents (interns) who were novice at intubation, defined as having performed ≤3 prior intubations on a patient, were eligible for inclusion in this study. The study took place during intern orientation, prior to Pediatric Advanced Life Support and Neonatal Resuscitation Program training. The study was approved by the hospital's Institutional Review Board.

At the initiation of the study, a survey was administered to the interns to document previous advanced life support certification and prior intubation experience with direct and videolaryngoscopy. Subjects then attended a thirty minute didactic session teaching the basics of pediatric airway anatomy and intubation procedures with both DL and GVL, followed by video and in-person expert modeling in small groups of the use of both laryngoscope techniques. For DL in an infant patient, subjects were instructed to position the tip of the laryngoscope blade under the epiglottis and to directly lift the large, floppy epiglottis covering the larynx. For GVL, subjects were instructed to identify the epiglottis and manipulate the videolaryngoscope to obtain the best glottic view. Subjects were not permitted to attempt intubation prior to the study.

The SimBaby high-fidelity simulator manikin (Laerdal Medical Corporation, NY, USA) was used for this study, which is representative of a 6-month-old infant [17]. Two intubation scenarios were used: (1) “normal intubation” with a standard airway and (2) “difficult intubation” with tongue edema and pharyngeal swelling, which has been noted previously to most consistently prolong time to intubation in the SimBaby [14]. Subjects were instructed to intubate SimBaby manikins in each scenario with both direct and videolaryngoscopy for a total of four intubations scenarios per subject. The sequence of scenarios and methods of intubation were randomized using a random number table to minimize the effect of learning throughout the study, and subjects were not informed which airway scenario would be next. Subjects did not receive any instruction during their intubation attempts.

Intubations were performed using a 3.0 mm endotracheal tube with a malleable stylet. The number 1 straight Miller laryngoscope blade was used for DL, and the GlideScope Cobalt AVL video baton 1-2 with GVL2 blade was used for GVL. For DL intubations, the endotracheal tube and stylet were configured in an arcuate shape, and for GVL intubations, the endotracheal tube and stylet were angled to 90 degrees to facilitate passage of the endotracheal tube [9–12, 18–21]. Airway manikin lubricant was applied to the endotracheal tube to decrease resistance between the endotracheal tube and the manikin airway. All materials were available at the bedside, and subjects did not have the option to choose different sized materials for intubation.

Subjects were instructed to ventilate the simulated infant in respiratory failure with a bag and mask and then to intubate the manikin. Intubation times were recorded from the time that the subject picked up the laryngoscope blade until clear lung inflation was achieved via the endotracheal tube with bag insufflation as captured by sensors in the simulator. Successful intubation was defined as successful insertion of the endotracheal tube between the vocal cords with clear lung inflation in ≤120 seconds [10, 15, 22–24]. Subjects were allowed to perform additional maneuvers at their discretion including repositioning and reintroducing the laryngoscope blade, repositioning the head or neck, digital laryngeal pressure, and modification of stylet tip angulation. 

Our primary outcomes were the time to successful intubation and the rate of successful intubation. Secondary outcomes included the number of intubation attempts, use of optimization maneuvers, self-reported view of the larynx, self-reported ease of use and satisfaction of the devices, and frequency of adverse events (esophageal or right mainstem intubation and dental trauma). Potential dental trauma was assessed using a previously described grading scale of pressure on the teeth or gums (0 = none, 1 = mild, 2 = moderate, and 3 = severe) [22, 24]. Subjects rated the Cormack and Lehane view of the larynx from grade I to IV [25] and the ease of use of each device for intubation on a visual analog scale (from 0 = difficult to 10 = easy). After completing the four intubation scenarios, subjects rated their overall satisfaction of the two laryngoscope techniques using the visual analog scale (from 0 to 10) and chose which device they would prefer for their next intubation.

### 2.2. Statistical Methods

Data were analyzed using SPSS Statistics (IBM, Armonk, NY, USA) and are described using mean and standard deviation for continuous data including time to intubation, dental trauma index, and subjective device ease of use score, and paired *t*-tests were used to compare continuous data. Descriptive statistical analyses were used for successful intubation, intubation attempts, use of optimization maneuvers, esophageal intubation, right mainstem intubation, and Cormack and Lehane glottis views. Nonparametric Wilcoxon signed ranks tests were used to compare categorical data. *P* < 0.05 was considered statistically significant, and a difference of 10 seconds in time to intubation was considered clinically significant. 

Sample size was based on the estimation of the time to intubation of 20 seconds with the standard Miller blade in the “normal intubation” scenario and an absolute variation of 10 seconds as a meaningful change based on previous studies with experienced physicians [14, 24]. We calculated a sample size of 10 participants using PASS software (NCSS, Kaysville, UT, USA) to have greater than 80% power to detect a minimal difference in time to intubation of 10 seconds assuming a type 1 error of 0.05. 

## 3. Results

Of the 29 interns approached, 25 interns fulfilled the inclusion criteria, and all 25 (100%) consented to participate in the study. Four interns were excluded for having performed >3 prior intubations on patients. Subjects' ages ranged from 24 to 29 years; 22 (88%) were female. Baseline characteristics of the residents are listed in Table 1. 

### 3.1. Normal Airway Scenario

The results for the normal airway scenario are shown in Table 2. There was no significant difference in subject performance in terms of successful intubation, time to successful intubation, number of intubation attempts, use of optimization maneuvers, esophageal intubation, right mainstem intubation, or subjective difficulty scores when either DL or GVL was used. The dental trauma index was significantly less when the GlideScope was used compared to the Miller laryngoscope (*P* = 0.013), and the Cormack and Lehane glottis view was significantly better with the GlideScope (*P* = 0.007). 

### 3.2. Difficult Airway Scenario

The results for the difficult airway scenario are shown in Table 3. Mean time for difficult airway intubation was significantly longer when GVL was used (*P* = 0.018). There was no significant difference in successful intubation, number of intubation attempts, use of optimization maneuvers, esophageal intubation, right mainstem intubation, or subjective difficulty scores between the two techniques. The dental trauma index was significantly less when using the GlideScope compared to the Miller laryngoscope (*P* = 0.013). In this scenario, Cormack and Lehane glottis views were better when the Miller laryngoscope was used (*P* = 0.046). 

### 3.3. Overall Ratings

Overall, there was a trend toward novice clinicians judging the GlideScope on a visual analog scale as easier to use compared to the Miller laryngoscope technique (mean 7.3 versus 6.4, *P* = NS). More interns chose the GlideScope than the Miller laryngoscope as the device of choice for their next intubation (17 versus 8).

## 4. Discussion

For novice clinicians, both the GlideScope and Miller laryngoscope methods of intubation were comparable in terms of time to intubation and the rate of successful intubations in a pediatric simulator model with normal airway. However, in the difficult airway scenario, the novice clinicians took significantly longer to intubate with GVL even though the overall rate of successful intubation was similar with both techniques. Yet overall, the subjects showed a two-to-one preference for GVL over the traditional DL. 

Our finding of novice clinician preference for GVL is in contrast to prior studies with experienced clinicians who preferred DL. In a study of anesthesiologists and pediatric intensivists, it was found that while there was no difference in time to intubation with GVL and DL, DL was rated higher in overall satisfaction, and the majority would choose DL in an emergency [14]. In another study, 85% of anesthesiologists preferred DL in an easy airway scenario and 30% preferred DL in a difficult airway scenario as well, even when the times to intubation were significantly shorter with GVL [26]. In a manikin study, emergency department physicians had a preference for DL in the normal airway and tongue edema scenarios and a preference for GVL only in the cervical spine immobilization scenario [10]. In an emergency department observational study, there was a strong preference for DL over GVL [27]. 

For experienced clinicians who have familiarity with DL, this preference for DL may be due to the personal bias of having complete control of the glottis view compared to GVL where there may be concern about the potential for obscuring of the glottis view with blood or secretions. In contrast, for novice clinicians in a simulated situation where these above-mentioned concerns are not apparent, the ease of visualizing the vocal cords as well as the superior view of the vocal cords with GVL may have led to a preference over DL. It may also be that younger clinicians who have grown up with video games may find the wider overall view of the larynx seen on the video monitor with GVL more natural and requiring less effort to obtain than the more restricted, direct view with DL. For novice clinicians, quick and easy visualization of the vocal cords and orientation of the airway anatomy with GVL may have provided them with comfort and reassurance so that even a lower grade laryngeal view and taking longer to insert the endotracheal tube in the difficult airway scenario, as has been demonstrated previously in pediatric patients [11], did not deter them from choosing GVL over DL for future use. 

We observed that the interns had difficulty maneuvering the endotracheal tube between the vocal cords with the GlideScope technique, as indicated by the longer duration of time to intubation in the difficult airway scenario. This delay in the insertion of the endotracheal tube with GVL has been noted by prior investigators and it appears to have arisen from the difficulty in insertion rather than due to difficulty in visualization [6, 8, 11, 13, 17, 20, 28]. For adults, there is a rigid stylet that is manufactured specifically for use with the GlideScope with appropriate angulation that follows the GlideScope blade and has been shown to contribute to higher first attempt and overall intubation success rates [29]. In contrast, pediatric intubations are performed with a malleable stylet which can be distorted easily and is less effective, thus potentially prolonging the time of intubation, especially for a novice. The malleable stylet used in our study was angled at the recommended 90 degree (“hockey stick”) formation for optimal insertion through the vocal cords [9, 18–21]. Despite this preparation of the stylet and endotracheal tube, it was difficult to maintain the correct angulation during intubation attempts due to the malleability and distortion of the stylet. This distortion of a malleable stylet has been noted more frequently in pediatric patients due to a smaller oropharynx and relatively larger tongue with the GlideScope blade taking up a larger proportion of the oropharynx, resulting in less room to maneuver the endotracheal tube [16, 21]. In addition, especially with tongue swelling and pharyngeal edema in the SimBaby, there was often a fair amount of resistance between the endotracheal tube and the manikin airway which complicated endotracheal insertion, and this has been noted by others as well in manikin studies [30]. Lastly, while easy to learn, the GlideScope does have a learning curve, and intubation success rates do improve with experience [31, 32]. 

The times to intubation in our study are longer than those in other studies in the literature, which have ranged from 10 to 50 seconds. This difference may be due to the fact that we studied novice clinicians rather than trained and experienced residents or physicians [3, 7, 9, 10, 22, 24, 26, 27, 33]. We also did not allow these novice interns to practice intubation with either method prior to the study. In addition, different studies have used different start and end points for time measurement, which may contribute to the variation in times to intubation. 

One advantage that has been consistently reported in GlideScope studies, including ours, is the reduction in dental trauma during intubations compared to DL [22–24]. Due to the angulation of the GlideScope, it is very easy to obtain the glottis view with minimal pressure on the anterior, superior portion of the mouth, and thus dental trauma is reduced. Another potential benefit of GVL is its usefulness in education since trainees can observe airway anatomy and intubations on the video monitor in real time and can be supervised while performing intubations. You et al. have shown the benefits of GVL in the education of novice clinicians [34]. 

Our study has several limitations. First of all, it is a manikin simulation study. Simulators are useful for teaching and assessing skills in novice persons without undue ethical or medical risks to patients. While the SimBaby is useful in this regard, it may not sufficiently mimic real-world clinical conditions. In addition, we did not allow any practice attempts with either intubating device prior to the start of the study since we were attempting to isolate the potential advantage of one device over another without prior experience. Practice attempts would allow subjects to familiarize themselves with the devices and intubation in general and would more closely resemble real clinical training situations. Furthermore, while subjects were blinded to the clinical scenario (normal versus difficult airway), they were not blinded to the device being used for intubation, which may have introduced bias. Lastly, while our intent was to use novice clinicians, there were some incoming residents who had experience with direct laryngoscopy, but none had intubated >3 patients, and none had near the suggested 57 intubations required for proficiency with a 90% success rate in direct laryngoscopy [35]. Additionally, since the subjects had some prior experience with direct laryngoscopy but not videolaryngoscopy, this may have biased participants towards direct laryngoscopy; however, the majority of subjects chose GlideScope videolaryngoscopy as the preferred method.

## 5. Conclusions

In conclusion, we found that for novice clinicians, GVL is not associated with improved intubation success rates or time to intubation in a pediatric simulator model of a normal airway scenario and had a longer time to intubation in a difficult airway scenario but with the same success rates. Despite the fact that it took longer for the novice physicians to intubate with GVL in the difficult airway scenario, their subjective assessment of ease of use of the devices after each scenario and overall was the same. Furthermore, novice physicians largely chose GVL over DL for their next intubation. The novice physicians likely placed a greater value on quick visualization of the vocal cords than that on the amount of time it took to intubate the manikins. In addition, GlideScope intubation may be safer with decreased dental trauma. More studies are needed to assess the clinical performance of GVL in pediatric situations, including real patient intubations and assessment of GlideScope performance for trainees over time.

## Figures and Tables

**Table 1:** Baseline characteristics of subjects (*N* = 25).

	*n* (%)
Previous advanced life support certification	6 (24)
Advanced cardiac life support (ACLS)	5 (20)
Pediatric advanced life support (PALS)	1 (4)
Previous intubation experience	
Patient (≤3 intubations)	8 (32)
Direct laryngoscopy	8 (32)
Videolaryngoscopy	0 (0)
Manikin	23 (92)
Direct laryngoscopy	23 (92)
Videolaryngoscopy	1 (4)

**Table 2:** Results for the normal airway scenario (*N* = 25).

	DL	GVL	*P *
Successful intubation*, *n* (%)	18 (72)	19 (76)	NS
Time to successful intubation*, mean (SD), sec	67.4 (27.9)	61.4 (26.5)	NS
Number of attempts, *n* (%)			
1	15 (60)	16 (64)	NS
2 or more	5 (20)	4 (16)	
Data not available	5 (20)	5 (20)	
Use of optimization maneuvers, *n* (%)	15 (60)	15 (60)	NS
Dental trauma index 0–3, mean (SD)	1.2 (0.8)	0.7 (0.8)	0.013
Esophageal intubation, *n* (%)	8 (32)	4 (16)	NS
Right mainstem intubation, *n* (%)	1 (4)	1 (4)	NS
Cormack and Lehane glottic view, *n* (%)			
I	14 (56)	23 (92)	0.007
II	11 (44)	2 (8)	
III/VI	0 (0)	0 (0)	
Ease of intubation score 0–10, mean (SD)	6.4 (1.5)	7.2 (1.9)	NS

*Up to 120 seconds.

DL: direct laryngoscopy; GVL: GlideScope videolaryngoscopy; NS: not significant.

**Table 3:** Results for the difficult airway scenario (*N* = 25).

	DL	GVL	*P *
Successful intubation*, *n* (%)	15 (60)	14 (56)	NS
Time to successful intubation*, mean (SD), sec	61.3 (19.3)	87.7 (26.9)	0.018
Number of attempts, *n* (%)			
1	13 (52)	11 (44)	NS
2 or more	5 (20)	5 (20)	
Data not available	7 (28)	9 (36)	
Use of optimization maneuvers, *n* (%)	19 (76)	19 (76)	NS
Dental trauma index 0–3, mean (SD)	2.0 (1.1)	1.1 (0.8)	0.013
Esophageal intubation, *n* (%)	4 (16)	5 (20)	NS
Right mainstem intubation, *n* (%)	1 (4)	1 (4)	NS
Cormack and Lehane glottic view, *n* (%)			
I	8 (32)	3 (12)	0.046
II	15 (60)	19 (76)	
III/VI	2 (8)	3 (12)	
Ease of intubation score 0–10, mean (SD)	5.7 (1.7)	5.3 (2.4)	NS

*Up to 120 seconds.

DL: direct laryngoscopy; GVL: GlideScope videolaryngoscopy; NS: not significant.

## References

[1] Bishop S, Clements P, Kale K, Tremlett MR (2009). Use of GlideScope ranger in the management of a child with treacher collins syndrome in a developing world setting. *Paediatric Anaesthesia*.

[2] Eaton J, Atiles R, Tuchman JB (2009). GlideScope for management of the difficult airway in a child with Beckwith-Wiedemann syndrome. *Paediatric Anaesthesia*.

[3] Karsli C, Armstrong J, John J (2010). A comparison between the GlideScope Videolaryngoscope and direct laryngoscope in paediatric patients with difficult airways—a pilot study. *Anaesthesia*.

[4] Lange M, Frommer M, Redel A (2009). Comparison of the GlideScope and Airtraq optical laryngoscopes in patients undergoing direct microlaryngoscopy. *Anaesthesia*.

[5] Milne AD, Dower AM, Hackmann T (2007). Airway management using the pediatric GlideScope in a child with Goldenhar syndrome and atypical plasma cholinesterase. *Paediatric Anaesthesia*.

[6] Cooper RM, Pacey JA, Bishop MJ (2005). Early clinical experience with a new videolaryngoscope (GlideScope) in 728 patients. *Canadian Journal of Anesthesia*.

[7] Kim J-T, Na H-S, Bae J-Y (2008). GlideScope videolaryngoscope: a randomized clinical trial in 203 paediatric patients. *British Journal of Anaesthesia*.

[8] Rai MR, Dering A, Verghese C (2005). The GlideScope system: a clinical assessment of performance. *Anaesthesia*.

[9] Sun DA, Warriner CB, Parsons DG, Klein R, Umedaly HS, Moult M (2005). The GlideScope Videolaryngoscope: randomized clinical trial in 200 patients. *British Journal of Anaesthesia*.

[10] Kim HJ, Chung SP, Park IC, Cho J, Lee HS, Park YS (2008). Comparison of the GlideScope videolaryngoscope and Macintosh laryngoscope in simulated tracheal intubation scenarios. *Emergency Medicine Journal*.

[11] Kaufmann J, Laschat M, Hellmich M, Wappler F (2013). A randomized controlled comparison of the Bonfils fiberscope and the GlideScope Cobalt AVL videolaryngoscope for visualization of the larynx and intubation of the trachea in infants and small children with normal airways. *Pediatric Anesthesia*.

[12] Redel A, Karademir F, Schlitterlau A (2009). Validation of the GlideScope videolaryngoscope in pediatric patients. *Paediatric Anaesthesia*.

[13] Fiadjoe JE, Gurnaney H, Dalesio N (2012). A prospective randomized equivalence trial of the GlideScope Cobalt videolaryngoscope to traditional direct laryngoscopy in neonates and infants. *Anesthesiology*.

[14] White M, Weale N, Nolan J, Sale S, Bayley G (2009). Comparison of the Cobalt GlideScope videolaryngoscope with conventional laryngoscopy in simulated normal and difficult infant airways. *Paediatric Anaesthesia*.

[15] Nouruzi-Sedeh P, Schumann M, Groeben H (2009). Laryngoscopy via macintosh blade versus GlideScope: success rate and time for endotracheal intubation in untrained medical personnel. *Anesthesiology*.

[16] Iacovidou N, Bassiakou E, Stroumpoulis K (2011). Conventional direct laryngoscopy versus videolaryngoscopy with the GlideScope: a neonatal manikin study with inexperienced intubators. *The American Journal of Perinatology*.

[17] Laerdal SimBaby directions for use. http://laerdalcdn.blob.core.windows.net/downloads/f1589/ABSTZAKJ/SimBaby-DfU-EN-5786-rev-B.pdf.

[18] Doyle DJ (2005). The GlideScope videolaryngoscope. *Anaesthesia*.

[19] Dupanović M, Isaacson SA, Borovčanin Ž (2010). Clinical comparison of two stylet angles for orotracheal intubation with the GlideScope videolaryngoscope. *Journal of Clinical Anesthesia*.

[20] Jones PM, Turkstra TP, Armstrong KP (2007). Effect of stylet angulation and endotracheal tube camber on time to intubation with the GlideScope. *Canadian Journal of Anesthesia*.

[21] Xue FS, Liu HP, Liu JH, Liao X, Zhang YM (2009). Facilitating endotracheal intubation using the GlideScope videolaryngoscope in children with difficult airways. *Paediatric Anaesthesia*.

[22] Fonte M, Oulego-Erroz I, Nadkarni L, Sánchez-Santos L, Iglesias-Vásquez A, Rodríguez-Núñez A (2011). A randomized comparison of the GlideScope videolaryngoscope to the standard laryngoscopy for intubation by pediatric residents in simulated easy and difficult infant airway scenarios. *Pediatric Emergency Care*.

[23] Malik MA, Hassett P, Carney J, Higgins BD, Harte BH, Laffey JG (2009). A comparison of the GlideScope, Pentax AWS, and Macintosh laryngoscopes when used by novice personnel: a manikin study. *Canadian Journal of Anesthesia*.

[24] Rodriguez-Nunez A, Oulego-Erroz I, Perez-Gay L, Cortinas-Diaz J (2010). Comparison of the GlideScope videolaryngoscope to the standard macintosh for intubation by pediatric residents in simulated child airway scenarios. *Pediatric Emergency Care*.

[25] Cormack RS, Lehane J (1984). Difficult tracheal intubation in obstetrics. *Anaesthesia*.

[26] Lim TJ, Lim Y, Liu EHC (2005). Evaluation of ease of intubation with the GlideScope or Macintosh laryngoscope by anaesthetists in simulated easy and difficult laryngoscopy. *Anaesthesia*.

[27] Platts-Mills TF, Campagne D, Chinnock B, Snowden B, Glickman LT, Hendey GW (2009). A comparison of GlideScope videolaryngoscopy versus direct laryngoscopy intubation in the emergency department. *Academic Emergency Medicine*.

[28] Tan BH, Liu EHC, Lim RTC, Liow LMH, Goy RWL (2009). Ease of intubation with the GlideScope or airway scope by novice operators in simulated easy and difficult airways—a manikin study. *Anaesthesia*.

[29] Sakles JC, Kalin L (2012). The effect of stylet choice on the success rate of intubation using the GlideScope videolaryngoscope in the emergency department. *Academic Emergency Medicine*.

[30] Benjamin FJ, Boon D, French RA (2006). An evaluation of the GlideScope, a new videolaryngoscope for difficult airways: a manikin study. *European Journal of Anaesthesiology*.

[31] Aziz MF, Healy D, Kheterpal S, Fu RF, Dillman D, Brambrink AM (2011). Routine clinical practice effectiveness of the GlideScope in difficult airway management: an analysis of 2,004 GlideScope intubations, complications, and failures from two institutions. *Anesthesiology*.

[32] Siu LWL, Mathieson E, Naik VN, Chandra D, Joo HS (2010). Patient- and operator-related factors associated with successful GlideScope intubations: a prospective observational study in 742 patients. *Anaesthesia and Intensive Care*.

[33] Nasim S, Maharaj CH, Malik MA, O’Donnell J, Higgins BD, Laffey JG (2009). Comparison of the GlideScope and Pentax AWS laryngoscopes to the Macintosh laryngoscope for use by advanced paramedics in easy and simulated difficult intubation. *BMC Emergency Medicine*.

[34] You JS, Park S, Chung SP, Park YS, Park JW (2009). The usefulness of the GlideScope videolaryngoscope in the education of conventional tracheal intubation for the novice. *Emergency Medicine Journal*.

[35] Konrad C, Schüpfer G, Wietlisbach M, Gerber H (1998). Learning manual skills in anesthesiology: is there a recommended number of cases for anesthetic procedures?. *Anesthesia and Analgesia*.

